# Rolly Protein (ROLP)-Epb4.1/3: A Potential Protein-Protein Interaction Relevant for the Maintenance of Cell Adhesion

**DOI:** 10.3390/ijms10052054

**Published:** 2009-05-12

**Authors:** Manuele Castelnuovo, Massimiliano Monticone, Sara Massone, Irene Vassallo, Federico Tortelli, Ranieri Cancedda, Aldo Pagano

**Affiliations:** 1 Oncology Biology and Genetics Department (DOBiG), University of Genoa, Italy; E-Mails: manuele.castelnuovo@istge.it (M.C.); sara.massone@fastwebnet.it (S.M.); irene.vassallo@email.it (I.V.); federico.tortelli@istge.it (F.T.); ranieri.cancedda@unige.it (R.C.); 2 National Institute for Cancer Research (IST), L.rgo R.Benzi 10, 16132, Genoa, Italy; 3 Advanced Biotechnology Center, L.rgo R.Benzi 10, 16132, Genoa, Italy; E-Mail: massimilianomonticone@istge.it (M.M.)

**Keywords:** cell adhesion, ROLP, Epb4.1/3, PSD-95, cell proliferation

## Abstract

We recently described Rolly Protein (ROLP), a small protein synthesized by substrate-adherent cells in a broad range of tissues. In a first set of experiments performed taking advantage of bone forming tibial cartilage as an experimental model we showed that ROLP transcription is associated to cells in an active proliferation state, whereas its downregulation is observed when cell proliferation decreases. Taking advantage of siRNA technology we also documented the expression modulation of some apoptosis-related genes in ROLP-silenced cells. In this work we search for the possible molecular interactors of ROLP by using both the antibody array approach as well as the co-immunoprecipitation approach. Results suggest the occurrence of an interaction of ROLP with Erythrocyte membrane Protein Band 4.1/3 (Epb4.1/3), an oncosuppressor downregulated in tumor development and in metastatic tissues; in addition we report experimental results that keep in line also with a potential interaction of ROLP with other PDZ-containing proteins. We also present experimental evidences supporting a role played by ROLP in cell adhesion thus supporting the existence of a biologically relevant link between ROLP and Epb4.1/3. We here suggest that ROLP might exert its biological role cooperating with Epb4.1/3, a protein that is involved in biological pathways that are often inhibited in tumor metastasis. Given the role of Epb4.1/3 in contrasting cancerogenesis we think that its cooperation with ROLP might be relevant in cancer studies and deserves further investigation.

## 1. Introduction

We recently characterized a novel gene encoding for a small protein that contains a predicted all-alpha secondary structure thus referred to as ROLP. ROLP is actively synthesized in a broad spectrum of tissues both in mouse as well as in chicken, but its highest level of expression is detected in bone forming tibial cartilage, in lung and in brain. In cartilage ROLP is expressed in proliferating cells, whereas its synthesis is dramatically down-regulated in differentiating and/or in resting cells. More in detail, in the tibial embryonic cartilage ROLP is actively synthesized by substrate-adherent fibroblast-like proliferating chondrocytes, whereas its protein signal is barely detectable when the cell proliferation rate decreases and the morphology of the cell changes to a somewhat more round shape and looses the adherence to the substrate undertaking the characteristic structural remodeling that occurs during chondrocyte differentiation. By coupling siRNA-based knock-down technology to global gene expression analysis (cDNA arrays) we identified an intriguing expression modulation of some apoptosis-related genes in ROLP-silenced cells, consistent with a role of ROLP in cell protection from apoptosis [1].

Modifications of the cell shape occur in a large number of important biological processes where they trigger several intracellular signaling pathways. Thus, cell scaffold changes consequent to variations of cell adhesion properties are at the basis of a wide spectrum of gene expression modulations. These processes are often triggered by protein super-complexes associated to the plasma membrane where they capture the first modifications of cell morphology and translate it into a message directed to the nucleus. In this context 4.1 and Dlg (Disc large) protein families represent key actors functioning as molecular linkers between the cytoskeleton and the plasma membrane [2–4]. These proteins bind to each other by the 4.1 binding motif (also present in the Dlg proteins) although they can interact with other partners by different domains such as Postsynaptic Density-95/Discs-large/Zonula occludens-1 (PDZ) domain or Src homology domain-3 (SH3) [5,6].

Here we show results obtained by experiments made in NIH 3T3 murine cells that suggest the possible interaction of ROLP with Epb4.1/3 (gene ID:13823), a protein of the 4.1 superfamily and with Dlg4/PSD-95 (NP_031890) and Integrin-alpha1 (NP_001028400); in addition, these experiments indicate the potential occurrence of a weak interaction of ROLP with other proteins associated with the cell membrane such as Rack 1 (CAI35106), p19S 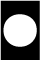 kp1 (NP_035673), Rab3 (NP_848805), Rab5 (NP_080163) and Rab11 (NP_059078). Interestingly all these possible partners of ROLP share functional and structural common hallmarks: i) they all contain one or more PDZ domains ii) they are localized at the plasma membrane level where they play a role in membrane recycling, membrane fusion in synaptic vesicle formation, and cell anchoring at membrane level.

Considering the possible association of ROLP with this group of proteins together with its tightly regulated expression during cell morphology changes occurring in the course of chondrocyte differentiation, we focused our study on a possible role played by ROLP in the maintenance of cell adhesion.

## 2. Experimental Section

### 2.1. Cell adhesion assay

This protocol is adapted from McClain *et al.* [18]. After 36 h of siRNA treatment cells were detached from the cell culture plate (polystyrene, cell culture treated) by trypsinization and plated at 150,000 cells/well in a 96-well tissue culture plate; Three hours after cell plating the multiwell plate was centrifuged twice in inverted position (well bottom placed up in a swing out rotor) at 1,000 rpm for 30 min. The cells still adherent to the wells were then processed in two different ways in independent experiments: 1) viable cells were evidenced by a MTT colorimetric method [7] or 2) they were washed twice with PBS, fixed in 3.7% paraformaldehyde in PBS, and stained with Methylene Blue. After the staining the cells were analyzed by digital imaging and cells were counted.

### 2.2. Real time quantitative RT-PCR

Dlg4/PSD-95 and Epb4.1/3 mRNAs were measured by Real-Time quantitative RT-PCR Sybr Green method, using PE ABI PRISM@ 7,700 Sequence Detection System (Perkin Elmer). The sequences of forward and reverse primers as designed by the Primer Express 1.5 software were: 1) Mouse ROLP 5′-TGAGTACAATGACATCAAGGATGTATCA-3′ and 5′-GATCAAAATCTGGATATAACTCCT TGGT-3′. 2) Dlg4/PSD-95: 5′-CACGAAGCTGGAGCAGGAG-3′ and 5′-GGCCTGAGAGGTCTTC GATG-3′. 3) Epb4.1/3: 5′-TGCAAAGTGACGCTTCTGGAT-3′ and 5′-GTCTACAAGCGTGTG AAACAG-3′. 4) Integrin alpha 1: ITGA1-F AGCAGCAGCAACCGGAAAC and ITGA1-RCA AAGGCGCTCAGGAGGAT. As endogenous control the expression of Glyceraldehyde 3 phosphate dehydrogenase (GAPDH) was examined by quantitative RT-PCR as described above. The mouse GAPDH primers were 5′-TGTGTCCGTCGTGGATCTG-3′ and 5′-GATGCCTGCTTCACCAC CTT-3′. Relative transcript levels were determined by the standard curve method following manufacturer's instructions.

### 2.3. Gene silencing

Target sequences for ROLP siRNAs were identified by detecting AA dinucleotides in ROLP mRNA sequence. The downstream 19 nucleotides were compared to the mouse genome database in order to select those with no homology to other genes. siRNAs were synthesized by using the Silencer siRNA Construction Kit (Ambion, UK) according to the manufacturer's instructions. The oligos used were: mROLP Antisense #13: 5′-AAGGAGTTATATCCAGATTTTCCTGTCTC-3′, mROLP Sense #13: 5′-AAAAAATCTGGATATAACTCCCCTGTCTC-3′. The oligos used to synthesize Epb4.1/3 silencers were: mEpb4.1/3 #1 Antisense 5′-AAGCTCTCTCAGAGATCATCCCCTGTCTC-3′ and mEpb4.1/3 #1 Sense 5′-AAGGATGATCTCTGAGAGAGCCCTGTCTC-3′; mEpb4.1/3 #2 Antisense 5′-AAG GAGTGGAGATTATGTTAGCCTGTCTC-3′ and mEpb4.1/3 #2 Sense 5′-AACTAACATAATCTC CACTCCCCTGTCTC-3′; mEpb4.1/3 #3 Antisense 5′-AAGGAGGTGCCCGTAGTCCACCCT GTCTC-3′ and mEpb4.1/3 #3 Sense 5′ AAGTGGACTACGGGCACCTCCCCTGTCTC-3′. The three double strand siRNAs were transfected in NIH 3T3 cells at different concentrations (0.1, 1, 10, 100 nM) by using Effectene Transfection Reagent (Qiagen) following manufacturer's instructions. ROLP expression was monitored (at mRNA level by real-time RT-PCR and at protein level by western blotting analysis) after 24, 48 and 72 h of siRNA treatment [1].

### 2.4. Cell culture and transient transfection

Murine fibroblast (NIH 3T3) and human neuroblastoma cells (SKNBE2 and SHSY5Y) were grown in a 5% CO_2_ incubator at 37 ºC in DMEM medium (Euroclone), containing 10% FBS (Gibco), 4 mM Glutamine, antibiotics. Subconfluent murine fibroblasts (3T3 cells), were transfected with Fugene HD (Roche) according to the manufacturer’s instructions.

### 2.5. Antibody array

Signal Tranduction AntibodyArrayTM (Cat #: HM3000, Hypromatrix, Inc.) was performed according to manufacturer’s instructions.

### 2.6. Immunoprecipitation

NIH-3T3 cells were grown overnight on a 6 cm dish and transfected with a specific silencer. After 30 h of silencing the cells were rinsed once with ice-cold PBS pH8, incubated with 1 mM chemical cross-linking dithiobissuccinimidyl dipropionate (DSP, Pierce Chemical Co., Rockford, IL) for 30 minutes at 25 °C and the reactions were quenched by adding 50 mM Tris-HCl, pH 7.5, for 15 minutes. Cells were then scraped, pelleted, and resuspended in 0.1% Triton X-100, 10 mM Tris, pH 7.5, 1 mM EDTA and protease inhibitor cocktail (mini-complete Roche). After 30 minutes on ice, samples were centrifugated at 11,000 g for 15 minutes and the supernatants quantified (Pierce) were used for the immunoprecipitation. Proteins were incubated for 2 hours at 25 °C with primary polyclonal antibody anti-ROLP and 20 mL Protein G-Sepharose, washed 3 times in ice-cold PBS pH 8. Samples were boiled for 5 minutes in Laemmli sample buffer plus 40 mM DTT and loaded on an SDS-PAGE gel.

### 2.7. Western blot analysis

Protein samples were quantified using a commercial quantification kit (Protein Assay, Bio-Rad) following manifacturer’s instructions. The samples were analyzed by 10% SDS PolyAcrylamide Gel Electrophoresis (SDS-PAGE) and transferred to a nitrocellulose membrane (Whatman, Inc.) or directly stained with Brilliant Blue G-colloidal concentrate (Sigma). The membranes were initially blocked by an incubation of 2 hours in Tris-buffered saline Tween 20 (TBST; 50 mM Tris-HCl, 150 mM NaCl, pH 7.5, 0.05% Tween 20) containing 5% non-fat dried milk. The blots were then incubated for 1 h with the primary antibody against ROLP protein, (see [1] for a detailed description of the antibody features). After washing with TBST membranes were incubated with peroxidase-conjugated anti-rabbit IgGs (A 0545, Sigma)(1:16,000) for 1 h at room temperature. After washing the reactive bands were revealed with ECL (Amersham Biosciences). The densitometric analysis of protein bands was performed taking advantage of ImageJ software system.

## 3. Results

### 3.1. Immunoprecipitation assay coupled to gene silencing technology in NIH3T3 cells suggests a possible interaction of ROLP with Epb4.1/3

**Figure 1.**
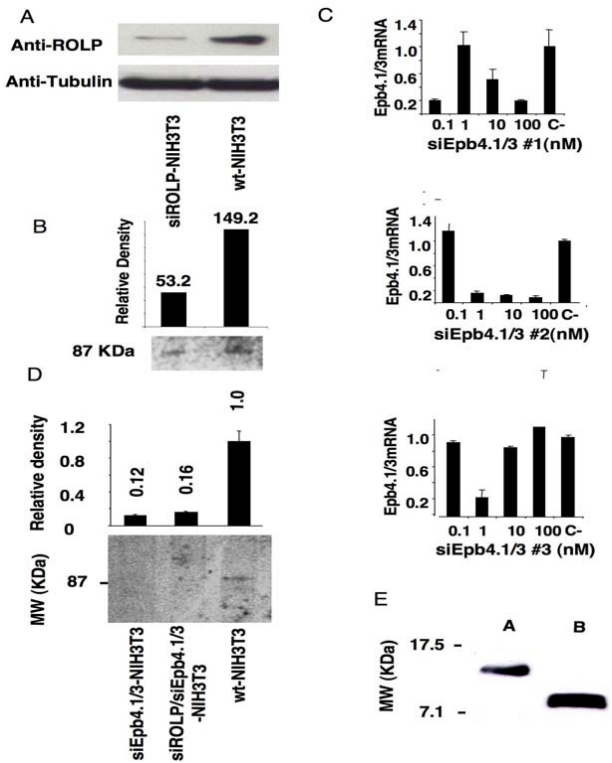
Analysis of Rolp protein-protein interactions. A) Western blot analysis indicating a strongly increased signal of ROLP in ROLP-silenced NIH3T3 cells with respect to their unsilenced counterpart. The unaltered concentration of β-Tubulin in the same samples is reported as control. B) Immunoprecipitation of ROLP in wild type NIH3T3 cells (wt-NIH3T3 lane) and in ROLP-silenced NIH3T3 (siRolp-NIH3T3 lane). A densitometric analysis of Epb4.1/3 protein bands in colloidal coomassie stained gel is reported. (C) The effects of different siEpb4.1/3 oligos (here referred to as #1, #2, #3) at different concentrations (0, 0.1, 1, 10, 100 nM) have been tested demonstrating that a treatment of 48 h with 100 mM #2 exerts the best silencing potential. The analysis of the mRNA expression was performed by Real time RT-PCR; the average of three determinations is reported. (D) Co-immunoprecipitation of ROLP and Epb4.1/3 in wild type NIH3T3 cells (wt-NIH3T3 lane), SiEpb4.1/3-NIH3T3 cells (siEpb4.1/3-NIH3T3 lane) and siEpb4.1/3-siROLP-NIH3T3 cells (siEpb4.1/3-siRolp-NIH3T3 lane); samples run in SDS PAGE were stained by colloidal coomassie. (E) Western blot analysis of mouse endogenous immunoprecipitated (lane A) and recombinant (lane B) ROLP.

In order to identify the biochemical pathway/s in which ROLP is involved we searched for its intracellular molecular interactor/s. By using a polyclonal ROLP-specific antiserum raised against the murine ROLP recombinant protein we co-immunoprecipitated ROLP together with its putative interactor/s in *wild type* NIH3T3 (hereafter referred to as *wt-*NIH3T3) as well as in ROLP-silenced NIH3T3 cells (hereafter referred to as siROLP-NIH3T3) [1]. In these experiments molecular interactions were stabilized by chemical cross-linking taking advantage of DSP (dithiobissuccinimidylpropionate), a lipofilic membrane-permeable ester used for both intracellular and intramembrane conjugation. Before performing the co-immunoprecipitation procedure we tested by western blot analysis (using anti-ROLP IgGs) the occurrence of ROLP silencing detecting a strongly decreased amount of ROLP in siROLP-NIH3T3 with respect to the unsilenced *wt*-NIH3T3 cell lysates (Figure 1A). Cells were then subsequently subjected to co-immunoprecipitation and the proteins were analyzed by SDS PAGE and revealed by colloidal coomassie staining.

A single band of about 87 KDa was observed in *wt*-NIH3T3 immunoprecipitation product, whereas the same protein signal was approximately three-fold weaker in the siROLP-NIH3T3 sample (149.2 *vs* 53.2), consistent with the 87 KDa polypeptide being a ROLP interactor (Figure 1B). The 87 KDa protein extracted from the gel was then analyzed by MALDI-TOF mass spectrometry in order to determine its individual identity. Mass spectrometry unambiguously identified the 87 KDa protein as Epb4.1/3, an oncosuppressor protein belonging to the Protein 4.1 superfamily. These molecules are localized at the interface between the cytoskeleton and the plasma membrane, where they play a significant role both in the maintenance of cell structural stability as well as in the transduction to the nucleus of signals triggered by cell shape modifications [8,9]. To strengthen the observation of a possible ROLP/Epb4.1/3 protein-protein interaction we repeated the co-immunoprecipitation in: 1) Epb4.1/3-silenced NIH3T3 cells (siEpb4.1/3-NIH3T3), 2) Epb4.1/3/ROLP-silenced cells (siEpb4.1/3-siROLP-NIH3T3), 3) control unsilenced *wt*-NIH3T3 cells. Results showed that neither in siEpb4.1/3-NIH3T3, nor in siROLP/siEpb4.1/3-NIH3T3 a protein product was immunoprecipitated by antiROLP IgGs whereas a band at 87 KDa was immunoprecipitated in NIH3T3-unsilenced cells (Figure 1C,D).

**Figure 2.**
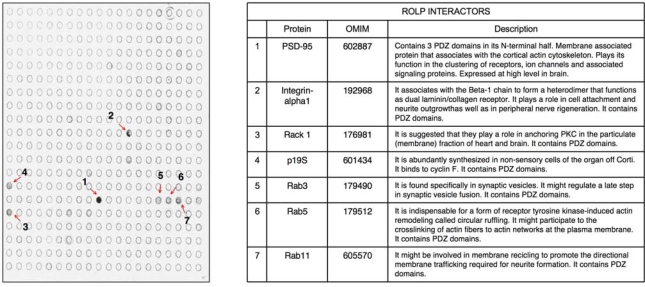
Antibody array analysis. The immunodetection with digoxigenin-labelled ROLP-specific IgGs revealed positive in several spots: PSD-95 (1), Integrin-alpha1 (2), Rack 1 (3), p19Skp1 (4), Rab3 (5), Rab5 (6), Rab11 (7). The OMIM code of each gene is reported together with the correspondent gene function.

Given the possible interaction of ROLP with a protein involved in cell signaling we decided to search for other possible functional partners acting in the same pathway not detected in the NIH3T3 co-immunoprecipitation experiments. To do this we challenged a cell signalling-specific antibody array that exposes 450 spots of cell signaling-associated antibodies with a *wt*-NIH3T3 cell lysate. In this experiment the proteins bound to ROLP (thus being its putative interactors) are captured by their specific antibodies immobilizing ROLP to specific spots correspondent to its interactors. The subsequent immunodetection with digoxigenin-labelled ROLP-specific IgGs leaded to the staining of a protein-specific signal in the position/s correspondent to the specific interactor/s. Results showed an intense signal correspondent to Dlg4/PSD95 (NP 031890), a PDZ-containing protein that acts in cell adhesion and in cell-cell contact maintenance (Figure 2).

Interestingly in the same experiment we detected a less intense signal in correspondence with few other spots compatible with a weak interaction of ROLP with six other proteins: Integrin-alpha1 (NP_001028400), Rack 1 (CAI35106), p19S 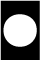 kp1 (NP_035673), Rab3 (NP_848805), Rab5 (NP_080163) and Rab11 (NP_059078) (Figure 2 and Table 1). Among these proteins Integrin-alpha 1 is of particular interest due to its key role in cell adhesion and to the intensity of its signal. Altogether the above results keep in line with the possible interaction of ROLP with PSD95 although its potential functional contact with Integrin-alpha1 and/or one of the other five proteins in a more physiological context cannot be excluded *a priori* by this approach.

### 3.2. The tissue expression profile of ROLP is compatible with its interaction with Epb4.1/3, PSD95 and Integrin-alpha1

**Figure 3.**
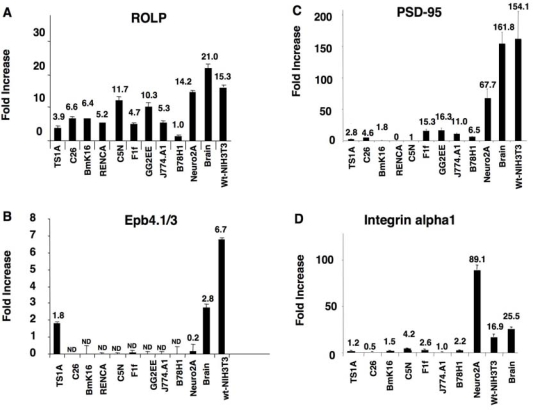
Tissue expression profile of ROLP, Epb4.1/3, PSD95 and Integrin-alpha1. (A, B, C, D) The analysis of the mRNA expression was performed by Real Time PCR. Results were normalized to an housekeeping gene expression (GAPDH). Each bar represents the mean ± SD.

As previous experiments suggested the association of ROLP to the prevention of apoptosis we tested its expression in a set of tumor cell lines, in brain and in NIH3T3 cells (a tissue and a cell line known to express ROLP at a high level). In the same samples we also measured the expression of Epb4.1/3, Integrin alpha 1 and PSD95. Results showed that ROLP is actively transcribed in almost all the tumor cell lines (with an expression peak in Neuro2A), in the brain and in *wt-*NIH3T3 (Figure 3A). To the contrary, (and consistently with its tumor suppressor activity [10]) Epb4.1/3 was found poorly expressed in all the tumor cell lines and actively transcribed by in Neuro2A cells, in brain and in *wt-*NIH3T3 cells (Figure 3B). Interestingly, PSD95 and Integrin-alpha1 were also highly expressed in Neuro2A, brain and *wt*-NIH3T3, thus presenting an expression profile similar to the one of Epb4.1/3 and parallel to the expression peaks of ROLP (Figure 3C,D). The coincident cell type-specificity of expression of these proteins is compatible with the occurrence of their biochemical interaction, although the rather ubiquitary expression of ROLP suggests that this protein might have other still unknown cell type-specific partners.

### 3.3. ROLP silencing prevents cell adhesion

It has been demonstrated that the over-expression of Epb4.1/3 leads to an increased cellular attachment [15], whereas PSD-95 is critical for the formation and the maintenance of synaptic junctions [6]; in addition, PSD-95 play a role as site-specific organizational center for integral membrane proteins and their downstream signaling molecules in the cortical cytoskeleton [16,17]. Considering these notions together with the subcellular localization of Epb4.1/3 and PSD95 at the plasma membrane and the fact that both proteins play an active role in cell signaling and adhesion we decided to test the possible participation of ROLP to these biological processes.

**Figure 4.**
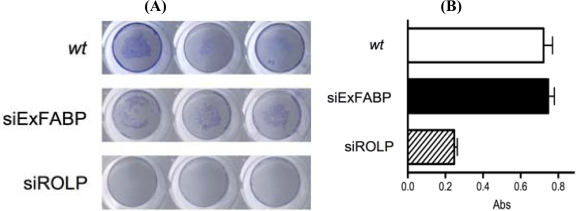
Cell adhesion properties assay of *wt*-NIH3T3, siEXFABP-NIH3T3 and siROLP-NIH3T3 cells. Viable, adherent cells were stained with methylene blue (A) or MTT (B) immediately after the adhesion essay. The bars represent the mean ± SD.

In our experimental approach the cell adhesion capacity of *wt*-NIH3T3 and siROLP-NIH3T3 cells was tested by a specific assay ([18] modified as described in material and methods). As control in the same experiment *wt-*NIH3T3 cells were transfected with an unrelated 21nt long siRNA that targets Ex-FABP, a chicken-specific protein (here referred to as siEx-FABP). Results showed that siROLP NIH3T3 cells have a decreased cell adhesion capacity that leads to the detachment of the cells from the substrate. As expected, in the same conditions both *wt-*NIH3T3 cells and siEx-FABP-NIH3T3 cells maintained their canonical adherence to the substrate (Figure 4A). A quantitative measurement of this phenomenon was obtained repeating the experiment in the same conditions and measuring by MTT [3-(4,5-dimethylthiazole-2-yl)-2,5-diphenyl tetrazolium bromide] test the relative amount of viable adherent cells. Again the results evidenced a significantly diminished number of adherent cells in siROLP-NIH3T3 cells (Figure 4B). Altogether these results keep in line with an active participation of ROLP in cell adhesion.

## 4. Discussion

In a previous paper we described ROLP as a novel protein with a predicted all-alpha secondary structures; we also reported that in chondrocytes ROLP is poorly synthesized by cells undergoing a differentiation program, whereas it is very abundant in those cells that remain in an active proliferation stage. Taking advantage of an *in vitro* model of chicken chondrocyte differentiation we previously documented that when chondrocytes undertake a differentiation program they modify their cell shape from a fibroblast-like substrate-adherent phenotype to a somehow round morphology associated to the hypertrophic fully differentiated stage [19]. In this context, our original observation that *in vitro* ROLP synthesis is restricted to substrate-adherent primary chondrocytes suggested its possible partecipation to the cell adhesion machinery. The data presented here indicate the molecular context in which ROLP exerts its role by the means of its protein-protein interaction partners and the possible involvement of these protein complexes in cell adhesion.

First, our data suggest that in NIH3T3 murine cells ROLP interacts with Epb4.1/3, a tumor suppressor protein which loss of function has been associated to the early events of meningioma development. Epb4.1/3 is a component of cytoskeleton-associated protein complexes that convey to the nucleus the information of the cell shape modification. The active expression of ROLP and Epb4.1/3 in both NIH3T3 as well as in neural crest-derived cells such as neuro2A, SHSY5Y and SKNBE neuroblastoma cell lines is compatible with their interaction suggesting that their cell type-specificity of synthesis is not incompatible with their functional contact. In the light of our data about the possible interaction of ROLP with Epb4.1/3 and considering their specific biological roles we here suggest that ROLP might cooperate with Epb4.1/3 in the transmission to the nucleus of cell shape modification-associated signals. In this context the experimental demonstration that ROLP-silenced murine fibroblasts loose the adherence to the substrate keeps in line with the possible participation of ROLP to cell adhesion and further support the potential occurrence of its functional cooperation with Epb4.1/3, which role in cell adhesion and proliferation control (through the activation of apoptosis) has been well established [15]. Therefore several functional similarities are shared by ROLP and Epb4.1/3: i) they affect cell proliferation ii) they play a role in cell adhesion and iii) the perturbation of their expression leads to a modulation of apoptosis-related genes and suggest a functional cooperation of these two polypeptides.

At a mechanistic level it is still unclear how ROLP could trigger the gene expression modulation that leads to the cell proliferation decrease, although it is interesting to note that this protein contains a leucine zipper motif, a structural feature most often associated to proteins with transcription regulation activity and thus might be able to play a direct role in the modulation of transcription of apoptosis-related genes. Altogether the above data suggest the investigation of ROLP in the biological processes in which Epb4.1/3 exerts its role.

In this work we also show data that prefigure a possible interaction of ROLP with Dlg4/PSD-95, Integrin-alpha1 and, at a lesser extent, with a set of different proteins involved in cell signaling. In regard to this it is interesting to note that, like Epb4.1/3, Dlg-related proteins are also associated with the cortical actin cytoskeleton where they play both structural and functional roles. In addition, it is well known that PSD95 binds the cytoplasmic COOH-termini of neuroligins, which are neuronal cell adhesion molecules that interact with neurexins and form intercellular junctions. Thus, the potential interaction of ROLP with Dlg4/PSD-95 (in addition to Epb4.1/3) would delineate a framework where ROLP might be functionally associated to one rather than the other of these two polypeptides in a cell type/cell stage-specific manner or that ROLP/Epb4.1/3/PSD95 might act in some conditions as part of the same protein super-complex. However, although the expression profile of PSD95 is compatible with its interaction with ROLP, further experiments are needed in order to confirm the occurrence of this interaction *in vivo*. At the present state one can reasonably hypothesize that: i) PSD95 and Epb4.1/3 interact with ROLp separately or ii) that PSD95 through its lysine-rich domain interacts with the membrane cytoskeletal linker protein 4.1 stabilizing the complex between ROLP and Epb4.1/3. Interestingly, PSD95 could also be involved in the modulation of a signal transduction cascade thanks to its GUK domain that binds GMP or GDP [2] thus being part of the same signaling network of Epb4.1/3. A further potential partner of ROLP, Integrin-alpha1, is of particular interest and deserves additional investigations as its role in cell adhesion keeps in line with a putative interaction with a protein involved in the same functional role such as ROLP.

## 5. Conclusions

In conclusion, the biochemical determinations that we report here strongly suggest that ROLP acts in cooperation with Epb4.1/3, a protein which expression is often abolished in tumor metastasis [20,21]. In this context this work suggests a possible way of investigation of ROLP function although further investigations are needed in order to elucidate structure, properties and functional traits of this novel protein complex. If this interaction will be further substantiated by other experimental approaches the pivotal role played by ROLP in cell shape modification signals as an adapter for different protein complexes will be confirmed. Given the involvement of Epb4.1/3 in cancerogenesis it is not unreasonable to speculate about a potential relevance of this novel protein complex for cancer studies.
